# Poincaré Plot Is Useful for Distinguishing Vasovagal Syncope From Postural Tachycardia Syndrome in Children

**DOI:** 10.3389/fped.2022.758100

**Published:** 2022-03-10

**Authors:** Piaoliu Yuan, Zhouhui Lian, Yuanyuan Wang, Yaru Wang, Chunyu Zhang, Junbao Du, Yaqian Huang, Ying Liao

**Affiliations:** ^1^Department of Pediatrics, Peking University First Hospital, Beijing, China; ^2^Wang Xuan Institute of Computer Science, Peking University, Beijing, China; ^3^Key Laboratory of Molecular Cardiovascular Sciences, Ministry of Education, Beijing, China

**Keywords:** Poincaré plot, vasovagal syncope, postural tachycardia syndrome, children, orthostatic intolerance, syncope, Holter, Lorenz-RR scatterplot

## Abstract

**Objectives:**

To explore the role of the Poincaré plot derived from a 24-hour Holter recording in distinguishing vasovagal syncope (VVS) from postural tachycardia syndrome (POTS) in pediatric patients.

**Materials and Methods:**

Pediatric patients with VVS or POTS, hospitalized in Peking University First Hospital between January 2012 and December 2018, were included in a derivation study. The transverse axis (T), longitudinal axis (L), T/L ratio, product T × L, distance between the origin and the proximal end of the longitudinal axis (pro-D), and distance between the origin and distal end of the longitudinal axis (dis-D) of the Poincaré plot were compared between the VVS and POTS groups, and the differential diagnostic performance of the above-mentioned graphic parameters was evaluated using receiver operating characteristic curve analysis. A validation study was conducted in pediatric patients hospitalized between January 2019 and December 2020.

**Results:**

In school-aged children, the T, L, T/L, T × L, and dis-D values of patients with VVS were greater than those of patients with POTS; in adolescents, the T, T/L, T × L, and pro-D values of patients with VVS were greater than those of patients with POTS. Using a T/L cut-off value of 0.3 to distinguish between the two diseases, the sensitivity and specificity were 91.0 and 90.5%, respectively, for the total participants; 91.6 and 88.9%, respectively, for the school-aged children; and 82.1 and 95.7%, respectively, for the adolescents. In the validation study, a T/L cut-off value of 0.3 yielded an accuracy, sensitivity, and specificity of 81.8, 87.2, and 77.6%, respectively, in the total participants; 76.5, 82.6, and 71.4%, respectively, in the school-aged children; and 89.2, 93.8, and 85.7%, respectively, in the adolescents, in distinguishing VVS from POTS validated by clinical diagnosis.

**Conclusions:**

The graphic parameters of the Poincaré plot are significantly different between VVS and POTS in pediatric patients, and the T/L of the Poincaré plot may be a useful measure to help differentiate VVS from POTS in children and adolescents.

## Introduction

Orthostatic intolerance (OI) refers to symptoms and signs that are elicited after orthostatism and relieved when lying down (1). Symptoms and signs include syncope, nausea, dizziness, palpitations, vomiting, blurred vision, pallor, and chest tightness (1). This condition is common in school-aged children and adolescents and affects learning and living in varying degrees (2). Vasovagal syncope (VVS) and postural tachycardia syndrome (POTS) are the most common underlying causes of OI in pediatric patients (2–4). However, the treatment strategies and prognoses differ significantly (2, 5). Therefore, differentiating VVS from POTS is very important in clinical practice (4, 6). Currently, the differential diagnosis between these two diseases is mainly based on the clinical features and haemodynamic responses during the head-up tilt test (HUTT) (2, 7). However, VVS and POTS sometimes have similar clinical features, such as recurrent syncope (2), making the diagnosis difficult (6); and the HUTT may cause discomfort and increase the psychological burden in children, and even lead to asystole in some cases (8–11). Further, the results of HUTT are influenced by the status of the patients and the experience of the operators, which limits its wide application in outpatient and primary clinics (6, 12). Therefore, a safe, easy-to-operate, and efficient indicator to assist the differentiation between VVS and POTS in clinical practice is required.

Previous studies have revealed that plasma hydrogen sulfide (13) and serum iron content (14) can be used as biomarkers to differentiate VVS from POTS in children. However, the gaseous molecule hydrogen sulfide is usually not stable in plasma and blood draw is an invasive procedure; thus, limiting their clinical applications. The autonomic nervous functional status of patients with VVS is different from that of patients with POTS (15–17). Previous studies showed that indicators of autonomic nervous functions, such as immediate changes in heart rate between the supine and the upright posture (15) and heart rate variability (HRV) frequency-domain indexes (18) can help in distinguishing VVS from POTS. However, the former is not visualized and involves a complicated measurement process using an electrocardiogram; furthermore, the efficiency of the latter in distinguishing VVS from POTS remains to be improved. Therefore, a better-visualized and more efficient method is urgently needed to distinguish VVS from POTS in children in clinical practice.

**Figure 1:**
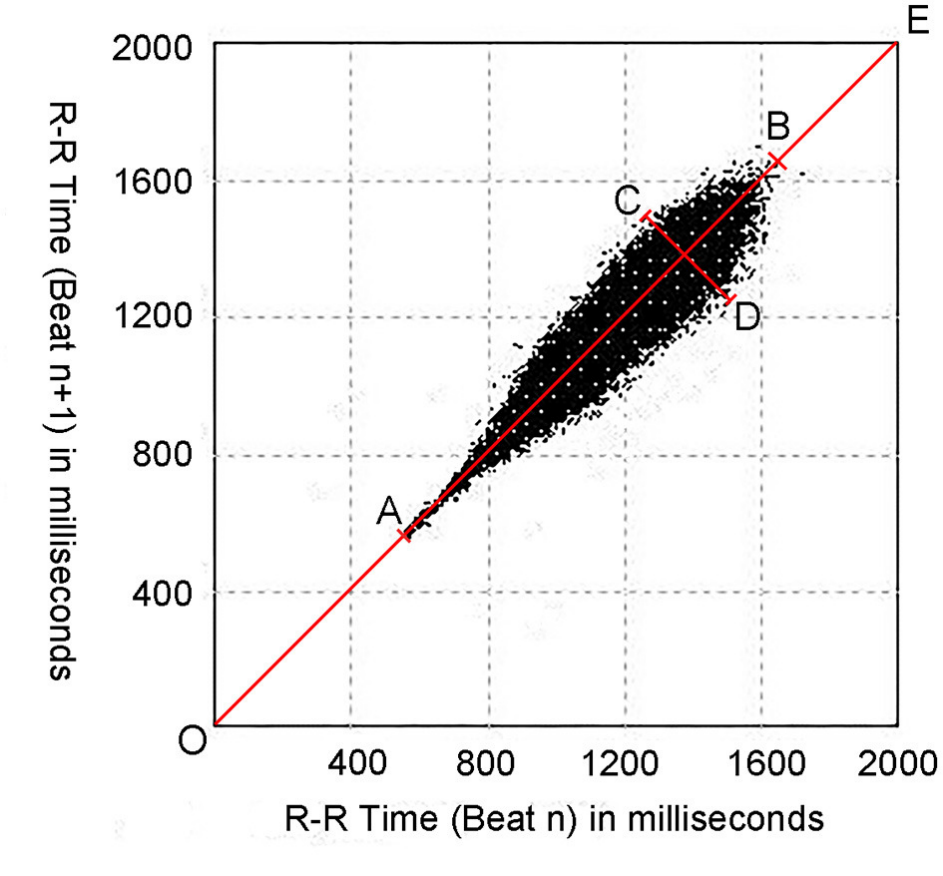
An example of the Poincaré plot from a 15-year-old female subject. Poincaré plots are constructed by plotting each RR interval (RR_n+1_) against the previous RR interval (RR_n_). OE represents the line of identity; AB represents the longitudinal axis (L); CD represents the transverse axis (T); OA represents the distance between the origin and the proximal end of the longitudinal axis (pro-D); and OB represents the distance between the origin and the distal end of the longitudinal axis (dis-D).

The Poincaré plot (Lorenz-RR scatterplot) is the most commonly used scatterplot based on a 24-hour (24-h) Holter electrocardiogram recording. It is an RR interval (i.e., time between adjacent heartbeats) scatter point set depicted in the rectangular plane coordinated system using adjacent RR intervals as the values of horizontal and vertical coordinates, respectively (Figure 1). The graphic characteristics of the Poincaré plot can comprehensively reflect the activity of the autonomic nervous system (19). For example, the longitudinal axis (L) of the Poincaré plot reflects the total heart rate distribution, which primarily reflects the sympathetic tone; while the transverse axis (T) of the Poincaré plot mainly reflects the instantaneous HRV, which primarily reflects the vagal tone (19–21). Therefore, the graphic parameters of the Poincaré plot are potential, non-invasive, visual, and easy-to-use indicators for differentiating VVS from POTS. The present study was designed to evaluate the role of graphic parameters of the Poincaré plot in distinguishing VVS from POTS in children. To the best of our knowledge, this was the first study to explore the role of the Poincaré plot in making a differential diagnosis of OI in children.

## Materials and Methods

### Participants

In the derivation study, 6–18-year-old children and adolescents with a definite diagnosis of VVS or POTS who were hospitalized in the Pediatrics Department, Peking University First Hospital, between January 2012 and December 2018 were included. They were classified into the VVS or POTS group based on the diagnosis at their discharge. In the validation study, 6–18-year-old children and adolescents who were hospitalized in the same hospital as the derivation study between January 2019 and December 2020 with a clinical diagnosis of VVS or POTS were enrolled. Boys aged 6–12 years and girls aged 6–11 years were classified as school-aged children; boys aged 13–18 years and girls aged 12–18 years were classified as adolescents (22).

#### Inclusion Criteria

(1) children aged 6–18 years old; (2) children diagnosed with VVS or POTS in the Pediatrics Department, Peking University First Hospital, between January 2012 and December 2020; and (3) participants with complete Poincaré plots of 24-h Holter recordings and hospitalization data.

#### Exclusion Criteria

(1) children with uncertain diagnoses, or VVS combined with POTS, or other diseases that may lead to transient loss of consciousness, including epilepsy, cardiogenic syncope, and psychogenic pseudo-syncope; (2) children who had received therapeutic interventions for VVS or POTS; (3) children with other diseases that would influence the graph of Poincaré plots, such as premature contraction, atrioventricular block, infection, hypertension, diabetes, anemia, hyperthyroidism/hypothyroidism, or peripheral neuropathy; or (4) children with incomplete (valid recording time <24 h) or low-quality Holter recordings.

The study was approved by the Ethics Committee of Peking University First Hospital [Number of Ethics Review: (2021) Science Research No. 150] and performed according to the Declaration of Helsinki. Since it was a retrospective study and all the data, which did not include any private information of the patients, were collected from the medical records system, the requirement for informed consent was waived by the Ethics Committee of Peking University First Hospital.

### Clinical Diagnostic Criteria

Pediatric VVS was diagnosed based on the following criteria: (1) presence of inducements, such as postural change from supine to upright position or long-term standing, mental stress or fear, or at a sultry environment; (2) presence of syncope as the main symptom; (3) a positive response in the HUTT; and (4) exclusion of other diseases that may manifest as transient loss of consciousness, for instance, cardiovascular, cerebrovascular, metabolic, and psychogenic diseases. The standards of a positive response during the HUTT include the following conditions: presence of syncope or presyncope with at least one of the following abnormalities: (1) significant reduction in blood pressure (BP) (systolic BP ≤ 80 mmHg or diastolic BP ≤ 50 mmHg, or a decline in mean BP of ≥25%); (2) significant decrease in heart rate (<75 bpm in 4–6-year-old participants; <65 bpm in 7–8-year-old participants; <60 bpm in pediatric patients > 8 years old); (3) electrocardiogram revealing sinus arrest; (4) sudden asystole or atrioventricular block lasting >3 s; and (5) the exclusion of orthostatic hypotension (decline in BP >20/10 mmHg during the first 3 min of the test) (2, 18, 23, 24). The haemodynamic types of responses during HUTT were classified as vasoinhibitory type, cardioinhibitory type, and mixed type (2). A vasoinhibitory type was characterized by a significant drop in BP without significant decrease in heart rate; a cardioinhibitory type was defined by a significant drop in heart rate without significant reduction in BP; and a mixed type referred to significant drops in both heart rate and BP during the HUTT (2).

The diagnostic criteria of pediatric POTS included the following: (1) inducements such as a postural change from supine to upright or long-term standing; (2) symptoms of OI elicited by standing upright; (3) standing test or HUTT that meets the standards of positive response; and (4) other causes of OI were excluded. A positive response for POTS in children during the standing test or HUTT was defined as follows: the heart rate is normal at a supine position and increases by ≥ 40 bpm and/or the highest heart rate reaches ≥ 130 bpm in participants aged 6–12 years; or ≥ 125 bpm in adolescents aged 13–18 years, within the first 10 min of the tests. No orthostatic hypotension is noticed during the test (2, 18, 23, 24).

### HUTT and Standing Test

#### HUTT

A quiet, warm, dimly lit environment with emergency equipment was prepared before performing the HUTT. All drugs that could affect autonomic tone and the cardiovascular system were stopped for ≥ 5 half-lives, and the participant was instructed to fast and refrain from drinking water for over 4 h prior to the test. After emptying the bladder, the participant was instructed to lay down on the examination bed (HUT-821, Beijing Juchi, China) and rest quietly for 10–30 min. BP, electrocardiogram data, and heart rate were continuously monitored. After the above indicators were stabilized, the patient was made to stand passively on a 60° inclined bed until the positive signs appeared or the 45-min test was completed (2).

#### Standing Test

The participant was first instructed to lay quietly for 10 min and then stay upright for 10 min. Heart rate, BP, and electrocardiogram of the participant were continuously monitored during the test (2).

### Twenty-Four-Hour Holter Monitoring and the Measurement and Calculation of Graphic Parameters

Participants were instructed to avoid food, drink, or medicine that could affect the activity of autonomic nervous system; strenuous exercise and emotional agitation were also avoided on the day of the examination and the day before. A Mortara Holter monitor (Mortara Instrument H3+^TM^/H12+^TM^, Milwaukee, Wisconsin USA) was used to record the 24-h electrocardiograms continuously, and the Mortara software (Mortara H-Scribe 7.0, Milwaukee, Wisconsin USA) was used to analyse the data and automatically construct as Poincaré plots.

As mentioned earlier, the Poincaré plot was constructed with a two-dimensional coordinate system using consecutive RR interval data derived from the 24-h Holter recording. The value on the *X*-axis of each point indicated the RR_n_ interval and the value on the *Y*-axis indicated the subsequent RR_n+1_ interval. The points [P (RR_n_, RR_n+1_)] were traced continuously in the coordinate system. When the points reached a certain number, a scatter-like graph was formed, and each 24-h Holter recording formed a Poincaré plot (25). Figure 1 details the quantitative measurement of the Poincaré plot's morphological properties. The line of identity (the 45° line in the coordinate system or the line OE in Figure 1) is the consulting line on which the two RR intervals of each point are equal, indicating that no heart rate acceleration or deceleration had occurred. The maximum length of the Poincaré plot along the line of identity was defined as L (the line AB in Figure 1), which stands for the total distribution of the heart rate; and the maximum width perpendicular to the line of identity was defined as T (the line CD in Figure 1), which represents the whole HRV (26–28).

The values of T, L, the distance between the origin and the proximal end of the longitudinal axis (pro-D), and the distance between the origin and the distal end of the longitudinal axis (dis-D) of the Poincaré plots were measured using Photoshop software (CS6, 13.0.1, Adobe Inc., CA, USA). To avoid errors caused by artifacts, only high-density points were measured (25) (Figure 1). The ratio of the T value to the L value (T/L) and the product of the two values (T × L) were then calculated.

All the Poincaré plots in the derivation and validation studies were measured by the same operator. Before these Poincaré plots were measured, the reproducibility and stability of the measurement method were tested. Two operators assessed the measurement of graphic parameters of Poincaré plots, respectively, on 20 participants; and one operator performed the same graphic parameters measurements twice for 20 participants in a one-week interval between the two measurements. The results obtained by the two operators as well as the two values of the measurement results from each participant were compared.

### Statistical Analysis

SPSS version 21.0 (IBM, New York, USA) was used for statistical analysis. Normality of continuous data was assessed using the Shapiro–Wilk test. Normally distributed data were summarized as mean ± SD, while non-normal data were summarized as median (interquartile range, IQR). Normally distributed data in the two groups were compared using an independent sample *t*-test, while non-normal data or ranked data were compared using the Mann–Whitney *U*-test. Reproducibility and stability of the measurement of the graphic parameters were assessed using the paired *t*-test. Categorical variables were expressed as *n* (%), and comparison between groups was performed using the chi-square test or Fisher's exact test. A *p*-value of < 0.05 indicated significant differences. Correlation of non-normally distributed data was assessed using the spearman linear correlation analysis, and the statistical variables were correlation coefficient *r*, 95% confidence interval (CI), and *p*-value. Differential diagnostic performance of graphic parameters of the Poincaré plot was assessed using receiver operating characteristic (ROC) curves. The area under the curve (AUC) represented the differential diagnostic value of the graphic parameters. When the 95% CI obtained did not include 0.5 and *p* < 0.05, the results were considered to be statistically significant. An AUC of 0.5–0.7 represented a low differential diagnostic value, an AUC of 0.7–0.9 represented a moderate differential diagnostic value, and an AUC of > 0.9 represented a high differential diagnostic value.

## Results

### Baseline Characteristics in Derivation Study

Based on the inclusion and exclusion criteria, 196 children were enrolled in the derivation study, including 122 children (56 boys and 66 girls; median age, 11.0 years old with a range of 6.0–17.0 years old) in the VVS group and 74 children (32 boys and 42 girls; median age, 13.0 years old with a range of 6.0–17.0 years old) in the POTS group. Participants in the VVS group were younger, had lower height and weight ranges, than those in the POTS group (*p* < 0.05) (Table 1). There was a statistically significant linear correlation between age and height (*r* = 0.796, 95% CI 0.726–0.853, *p* < 0.001) as well as between age and weight (*r* = 0.684, 95% CI 0.596–0.754, *p* < 0.001) in the derivation study. The most common symptom among children with VVS was syncope with a frequency less than once per month, while the most common manifestations among children with POTS were chronic, non-episodic symptoms of OI (*p* < 0.05) (Table 1). All patients in VVS group underwent HUTT and had positive responses meeting the diagnostic criteria of pediatric VVS with a median positive reaction time after tilt of 17.0 min (range, 4.0–45.0 min). Overall, 66 of the 74 patients in the POTS group underwent HUTT, among whom 57 had positive responses meeting the diagnostic criteria of pediatric POTS; and the remaining 17 patients who had a negative response or did not undergo HUTT showed a positive response of POTS during the standing test (Table 1). The maximum heart rate of the children in the POTS group was (122 ± 12) bpm and the median increase of heart rate from the recumbent to orthostatic position was 45 bpm (IQR, 40 bpm −51 bpm) during the standing test or within the first 10 min of HUTT. There were no significant differences in sex ratio, body mass index (BMI), supine heart rate, supine systolic BP, and supine diastolic BP between the two groups.

**Table 1:** Comparison of baseline characteristics between VVS and POTS cases in derivation study.

**Variables**	**VVS (*n* = 122)**	**POTS (*n* = 74)**	***t/Z/*χ^2^^a^**	***p***
Female, *n* (%)	66 (54.1%)	42 (56.8%)	0.132	0.717
Age, year	11.0 (9.0–13.0)	13.0 (11.0–14.0)	−4.681	**<0.001**
Height, cm	151.0 (140.0–162.0)	160.5 (152.8–168.6)	−4.434	**<0.001**
Weight, kg	41.8 (32.0–50.8)	48.8 (40.0–56.1)	−3.008	**0.003**
BMI, kg/m^2^	17.9 (16.0–21.0)	18.3 (16.9–20.3)	−0.874	0.382
Symptoms, *n* (%)			199.555	**<0.001**
Syncope^b^, *n* (%)	108 (88.5%)	0	–	–
With prodromal symptoms/sign^c^	102 (94.4%)	0	–	–
Chronic non-episodic OI, *n* (%)^d^	0	49 (66.2%)	–	–
With ≥ 5 symptoms	0	18 (36.7%)	–	–
Syncope + chronic non-episodic OI, *n* (%)	14 (11.5%)	25 (33.8%)	–	–
Frequency of symptoms, *n* (%)			−8.169	**<0.001**
<1 episode/month	93 (76.2%)	15 (20.3%)	–	–
2–4 episodes/month	15 (12.3%)	15 (20.3%)	–	–
2−7 episodes/week	9 (7.4%)	14 (18.9%)	–	–
>1 episode/day	5 (4.1%)	30 (40.5%)	–	–
Supine heart rate, bpm	75 (69–83)	75 (68–82)	−0.428	0.669
Supine systolic BP, mmHg	104.0 (98.0–110.0)	106.0 (99.0–116.0)	−1.742	0.082
Supine diastolic BP, mmHg	62.1 ± 8.2	62.6 ± 7.4	−0.442	0.659
Hemodynamic types, *n* (%)				
VVS-vasoinhibitory type	70 (57.4%)	0	–	–
VVS-cardioinhibitory type	5 (4.1%)	0	–	–
VVS-mixed type	47 (38.5%)	0	–	–
POTS	0	57 (77.0%)	–	–
Negative response	0	9 (12.2%)^e^	–	–
No HUTT results	0	8 (10.8%)^e^	–	–

*Data are n (%), mean ± SD, or median (interquartile range). The **bold** values are < 0.05, which indicates significant differences. BMI, body mass index; BP, blood pressure; HUTT, head-up tilt test; OI, orthostatic intolerance; POTS, postural tachycardia syndrome; VVS, vasovagal syncope*.

a *Variable “female” was compared using chi-square test; variables “age”, “height”, “weight”, “BMI”, “frequency of symptoms”, “supine heart rate”, and “supine systolic BP” were compared using the Mann–Whitney U-test; variable “symptoms” was compared using Fisher's exact test; and variable “supine diastolic BP” was compared using independent sample t-test*.

b *Syncope only, no symptoms of chronic OI were recorded*.

c *Prodromal symptoms/signs referred to the symptoms or signs of discomfort before the onset of loss of consciousness, such as dizziness, chest tightness, headache, visual difficulties, pallor, and sweating (2)*.

d *Chronic non-episodic OI included light-headedness, headache, visual difficulties, hearing changes, chest tightness, palpitations, gastrointestinal symptoms (nausea, abdominal pain, and emesis), hand tremors, numbness in extremities, muscle or joint pain, profuse sweating, inattention, and fatigue (29)*.

e *Patients with POTS who had a negative response or did not undergo HUTT showed a positive response of POTS during the standing test*.

As the shape of the Poincaré plot changes with the age of children, the participants enrolled in the derivation study were divided into two age stratifications (school age and adolescence). The age-stratified analysis revealed no distinct differences in sex ratio, age, height, weight, BMI, supine heart rate, supine systolic BP, and supine diastolic BP between the VVS and the POTS groups within the two age stratifications (*p* > 0.05) (Table 2). No remarkable differences were found between the values measured by the two operators (*p* > 0.05) (Supplementary Table 1), indicating that the measuring method was reproducible; no significant differences were found between the values measured 1 week apart by the same operator (Supplementary Table 2), indicating that the measuring method was stable.

**Table 2:** Baseline characteristics of VVS and POTS cases in age-stratified analysis in derivation study.

**Age stratification**	**School age**				**Adolescence**			
**Groups**	**VVS (*n* = 83)**	**POTS (*n* = 27)**	***t/Z/*χ^2^^a^**	***p***	**VVS (*n* = 39)**	**POTS (*n* = 47)**	***t/Z/*χ^2^^a^**	***p***
Female, *n* (%)	39 (47.0%)	13 (48.1%)	0.011	0.916	27 (69.2%)	29 (61.7%)	0.532	0.466
Age, year	10.0 (8.0–11.0)	10.0 (9.0–11.0)	−1.381	0.167	13.0 (13.0–15.0)	14.0 (13.0–15.0)	−1.683	0.092
Height, cm	144.0 (137.0–151.0)	151.0 (137.0–158.0)	−1.894	0.058	164.0 (155.0–168.0)	166.0 (160.0–171.0)	−1.576	0.115
Weight, kg	36.1 (30.0–44.0)	40.0 (32.0–50.0)	−1.327	0.185	50.1 (46.0–59.0)	52.5 (45.3–57.4)	−0.013	0.990
BMI, kg/m^2^	17.0 (15.8–20.1)	17.5 (16.0–20.5)	−0.688	0.492	19.4 (17.9–21.5)	19.0 (17.4–20.3)	−1.141	0.254
Symptoms, *n* (%)			93.458	**<0.001**			82.287	**<0.001**
Syncope^b^, *n* (%)	76 (91.6%)	0	–	–	32 (82.1%)	0	–	–
With prodromal symptoms/sign^c^	70 (92.1%)	0	–	–	32 (100%)	0	–	–
Chronic non-episodic OI, *n* (%)^d^	0	18 (66.7%)	–	–	0	31 (66.0%)	–	–
With ≥ 5 symptoms	0	7 (38.9%)	–	–	0	11 (35.5%)	–	–
Syncope + non-episodic OI, *n* (%)	7 (8.4%)	9 (33.3%)	–	–	7 (17.9%)	16 (34.0%)	–	–
Frequency of symptoms, *n* (%)			−4.887	**<0.001**			−5.336	**<0.001**
<1 episode/month	66 (79.5%)	9 (33.3%)	–	–	27 (69.2%)	6 (12.8%)	–	–
2–4 episodes/month	10 (12.1%)	6 (22.2%)	–	–	5 (12.8%)	9 (19.1%)	–	–
2–7 episodes/week	5 (6.0%)	2 (7.4%)	–	–	4 (10.3%)	12 (25.5%)	–	–
1 episode/day	2 (2.4%)	10 (37.1%)	–	–	3 (7.7%)	20 (42.6%)	–	–
Supine heart rate, bpm	77 (70–86)	78 (71–84)	−0.528	0.597	72 (62–76)	73 (64–78)	−0.825	0.409
Supine systolic BP, mmHg	104.0 (97.0–108.0)	101.0 (97.0–106.0)	−0.435	0.664	106.6 ± 10.0	108.9 ± 8.9	−1.142	0.257
Supine diastolic BP, mmHg	61.6 ± 8.0	61.5 ± 7.9	0.089	0.929	63.0 ± 8.8	63.2 ± 7.1	−0.139	0.890
Hemodynamic types, *n* (%)								
VVS-vasoinhibitory type	49 (59.0%)	0	–	–	21 (53.8%)	0	–	–
VVS-cardioinhibitory type	2 (2.4%)	0	–	–	3 (7.7%)	0	–	–
VVS-mixed type	32 (38.6%)	0	–	–	15 (38.5%)	0	–	–
POTS	0	21 (77.8%)	–	–	0	36 (76.6%)	–	–
egative response	0	3 (11.1%)^e^	–	–	0	6 (12.8%)^e^	–	–
No HUTT results	0	3 (11.1%)^e^	–	–	0	5 (10.6%)^e^	–	–

*Data are n (%), mean ± SD, or median (interquartile range). The **bold** values are < 0.05, which indicates significant differences. BMI, body mass index; BP, blood pressure; HUTT, head-up tilt test; OI, orthostatic intolerance; POTS, postural tachycardia syndrome; VVS, vasovagal syncope. A p-value of < 0.05 indicated significant differences*.

a *Variable ‘female' was compared using chi-square test; variables ‘age', ‘height', ‘weight', ‘BMI', ‘frequency of symptoms', ‘supine heart rate', and ‘supine systolic BP' in school-age children were compared using the Mann–Whitney U-test; variable ‘symptoms' was compared using Fisher's exact test; and variable ‘supine systolic BP' in adolescents and ‘supine diastolic BP' were compared using independent sample t-test*.

b *Syncope only, no symptoms of chronic OI were recorded*.

c *Prodromal symptoms/signs referred to the symptoms or signs of discomfort before the onset of loss of consciousness, such as dizziness, chest tightness, headache, visual difficulties, pallor, and sweating (2)*.

d *Chronic non-episodic OI included lightheadedness, headache, visual difficulties, hearing changes, chest tightness, palpitations, gastrointestinal symptoms (nausea, abdominal pain, and emesis), hand tremors, numbness in extremities, muscle or joint pain, profuse sweating, inattention, and fatigue (29)*.

e *Patients with POTS who had a negative response or did not undergo HUTT showed a positive response of POTS during the standing test*.

### Comparisons of Graphic Parameters of Poincaré Plot Between VVS and POTS Groups in Derivation Study

**Figure 2:**
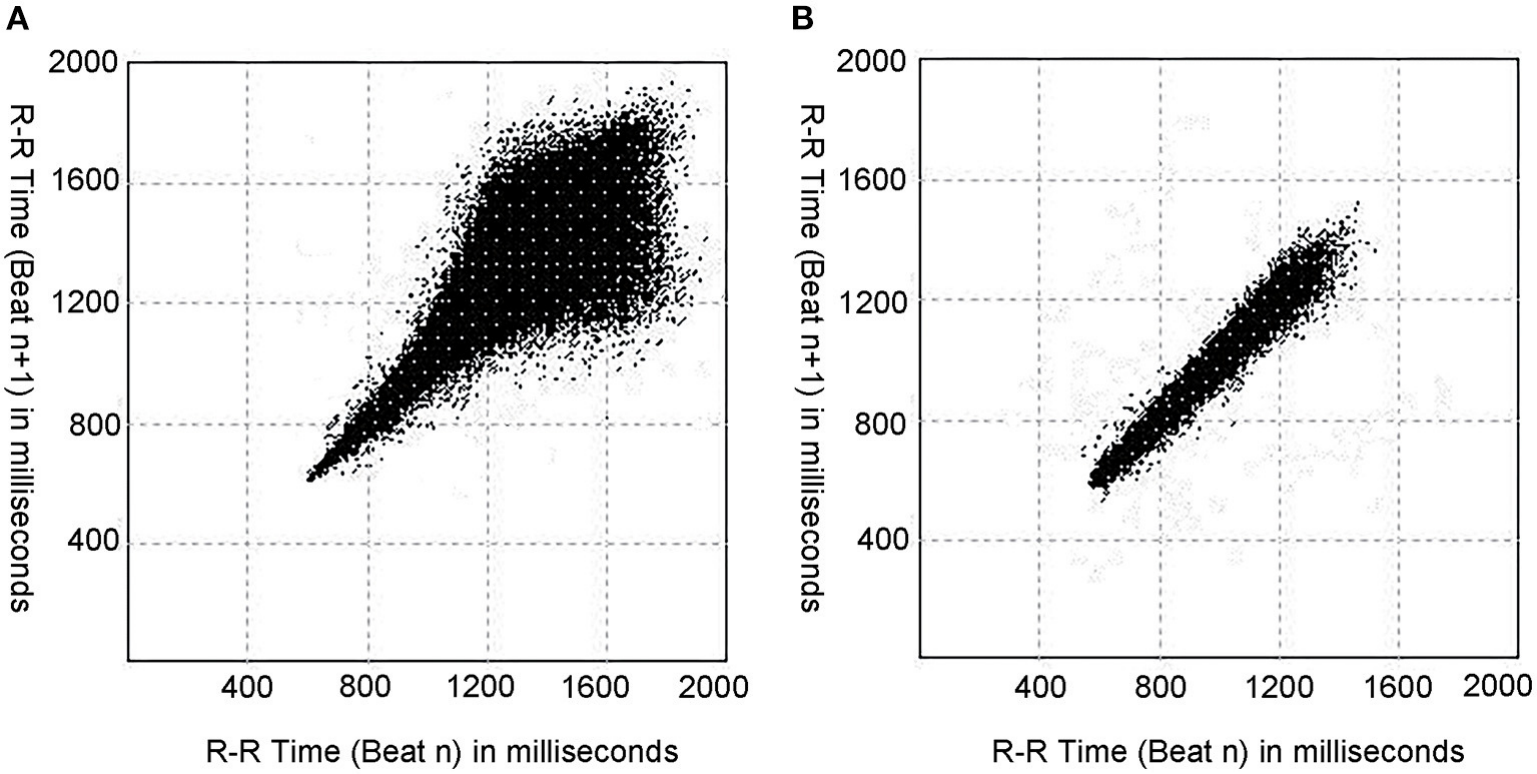
Typical Poincaré plots in a patient with VVS and a patient with POTS. **(A)** The Poincaré plot of a patient with VVS (male, 11 years old). **(B)** The Poincaré plot of a patient with POTS (male, 11 years old). As shown, the patient with VVS and the patient with POTS have different typical graphics of Poincaré plot, and the Poincaré plot of the patient with VVS tend to be tennis racket-shaped, while that of the patient with POTS tend to be baseball bat-shaped. VVS, vasovagal syncope; POTS, postural tachycardia syndrome.

In the school-aged children, the T, L, T/L, T × L, and dis-D values of Poincaré plot of patients with VVS were greater than those of patients with POTS (*p* < 0.001 for T, T/L, and T × L; *p* = 0.032 for L; *p* = 0.007 for dis-D); while in the adolescents, the T, T/L, T × L, and pro-D values of patients with VVS were greater than those of patients with POTS (*p* < 0.001 for T, T/L, and T × L; *p* = 0.004 for pro-D). No significant differences were found in the other graphic parameters of the Poincaré plot between the VVS and POTS groups (Table 3; Figure 2)

**Table 3:** Comparison of graphic parameters of Poincaré plot between VVS and POTS groups in derivation study.

**Age stratification**	**School age**				**Adolescence**			
**Groups**	**VVS (*n* = 83)**	**POTS (*n* = 27)**	***t/Z* ^a^**	***p***	**VVS (*n* = 39)**	**POTS (*n* = 47)**	***t/Z* ^a^**	***p***
T, ms	673.9 ± 186.7	381.8 ± 117.1	9.587	**<0.001**	633.0 ± 187.4	394.7 ± 105.9	7.063	**<0.001**
L, ms	1564.0 ± 244.4	1451.0 ± 199.1	2.177	**0.032**	1616.9 ± 224.1	1581.5 ± 229.8	0.721	0.473
T/L	0.415 (0.359–0.484)	0.256 (0.220–0.285)	−6.956	**<0.001**	0.390 ± 0.093	0.248 ± 0.046	8.636	**<0.001**
T × L, s^2^	0.991 (0.788–1.420)	0.556 (0.393–0.765)	−5.581	**<0.001**	0.956 (0.760–1.323)	0.586 (0.440–0.827)	−4.888	**<0.001**
pro-D, ms	788.0 ± 76.1	759.9 ± 66.9	1.714	0.089	850.6 (799.3–901.8)	778.8 (737.8–860.8)	−2.887	**0.004**
dis-D, ms	2352.0 ± 241.9	2210.9 ± 188.4	2.767	**0.007**	2477.3 ± 248.6	2379.5 ± 244.7	1.831	0.071

*Data are mean ± SD or median (interquartile range). The **bold** values are < 0.05, which indicates significant differences. Dis-D, the distance between the origin and the distal end of the longitudinal axis; L, longitudinal axis; ms, millisecond; POTS, postural tachycardia syndrome; pro-D, the distance between the origin and the proximal end of the longitudinal axis; s, second; T, transverse axis; T/L, the ratio of transverse axis value to longitudinal axis value; T × L, the product of transverse axis value and longitudinal axis value; VVS, vasovagal syncope*.

a *Normally distributed data were compared using the independent sample t-test, and non-normal data were compared using the Mann–Whitney U-test*.

### Evaluation of Differential Diagnostic Performance of Poincaré Plot for Distinguishing VVS From POTS in Derivation Study

**Figure 3:**
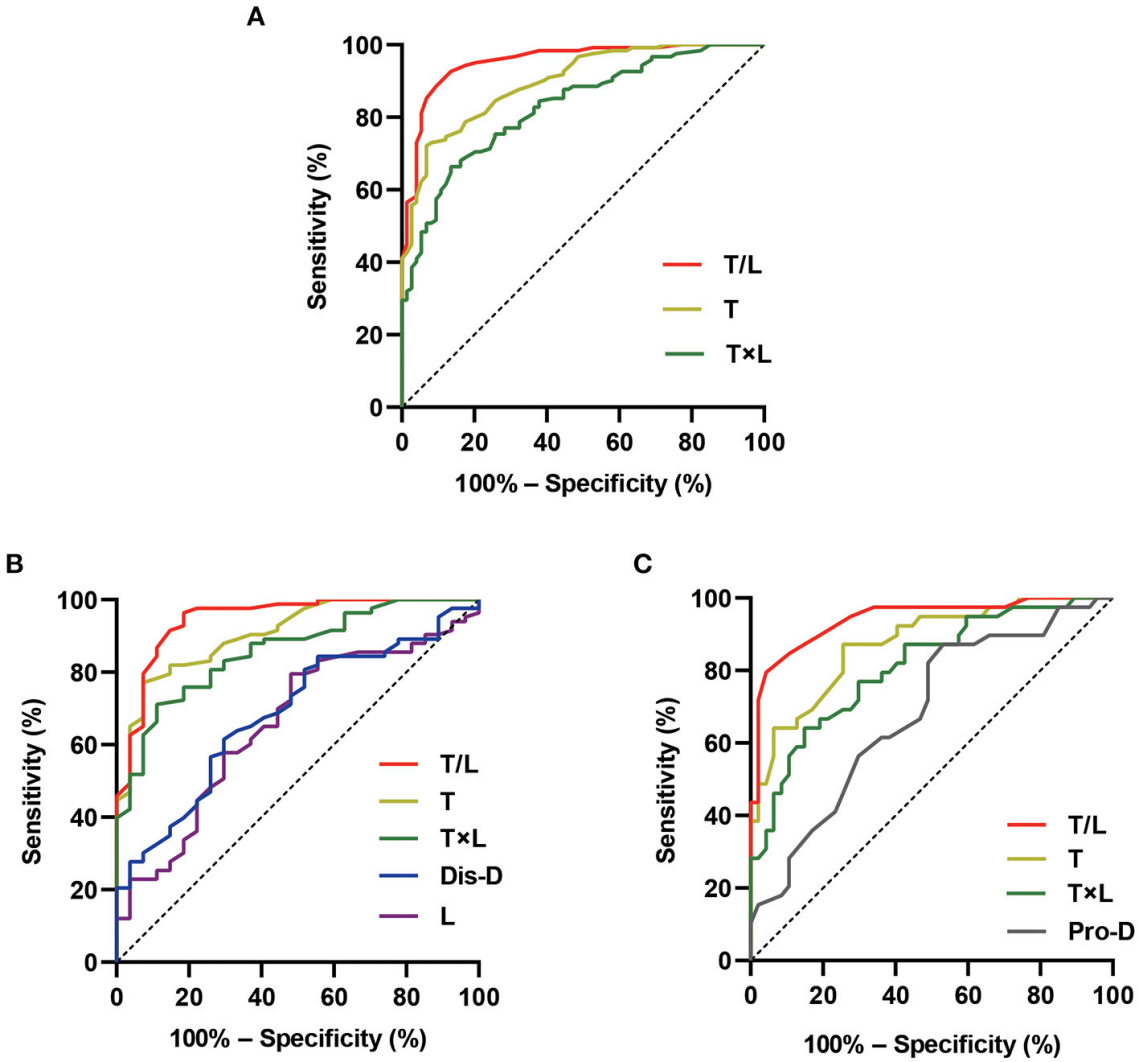
Differential diagnostic performance of the Poincaré plot. The ROC curves show the differential diagnostic performance of the graphic parameters of Poincaré plot in distinguishing VVS from POTS in the total participants **(A)**, school-aged children **(B)**, and adolescents **(C)** in the derivation study. The *Y*-axis shows the sensitivity to identify VVS; the *X*-axis shows the false-positive rate [100%–specificity (%)]. The black straight dotted line located in the chart at 45° to the coordinate axes represents the equivalence between the sensitivity and the false-positive rate (that is, no differential diagnostic value). The areas under the color curves represent the differential diagnostic performance of the graphic parameters of Poincaré plot. The larger the area under the curve, the better the differential diagnostic performance. Dis-D, the distance between the origin and the distal end of the longitudinal axis; L, longitudinal axis; POTS, postural tachycardia syndrome; pro-D, the distance between the origin and the proximal end of the longitudinal axis; ROC, receiver operating characteristic; T, transverse axis; T/L, the ratio of transverse axis value to longitudinal axis value; T × L, the product of transverse axis value and longitudinal axis value; VVS, vasovagal syncope.

The discriminatory performance of the parameters that were significantly different between the patients with VVS and those with POTS in the abovementioned univariate analysis was assessed using the ROC curves. The results are shown in Figure 3 and Table 4. All the analyzed parameters had statistical value for distinguishing between VVS and POTS (in total participants, *p* < 0.001 for T/L, T, and T × L; in the school-aged children, *p* < 0.001 for T/L, T, and T × L; *p* = 0.004 for dis-D; *p* = 0.015 for L; in the adolescents, *p* < 0.001 for T/L, T, and T × L; *p* = 0.004 for pro-D), among which T/L showed the maximum AUC, regardless of whether it was in the total participants, or either age stratification. In all the participants, the cut-off values of T, T/L, and T × L set at 538.0 ms, 0.3, and 0.87 s^2^, yielded sensitivities of 72.1, 91.0, and 66.4%, and specificities of 93.2, 90.5, and 86.5%, respectively, to differentiate VVS from POTS. In the school-aged children, the cut-off values of T, L, T/L, T × L, and dis-D set at 527.8 ms, 1,398.8 ms, 0.3, 0.82 s^2^, and 2280.2 ms, yielded sensitivities of 77.1, 79.5, 91.6, 71.1, and 61.4%, and specificities of 92.6, 51.9, 88.9, 88.9, and 70.4%, respectively, to differentiate VVS from POTS. In the adolescents, the cut-off values of T, T/L, T × L, and pro-D set at 466.3 ms, 0.3, 0.87 s^2^, and 773.7 ms, yielded sensitivities of 87.2, 82.1, 64.1, and 87.2%, and specificities of 74.5, 95.7, 85.1, and 46.8%, respectively, to differentiate VVS from POTS.

**Table 4:** Differential diagnostic performance of Poincaré plot in distinguishing VVS from POTS in derivation study.

**Age stratification**	**Parameters**	**AUC**	***p***	**95% CI**	**Cut-off value**	**Sensitivity (%)**	**Specificity (%)**
School age and adolescence	T/L	0.953	<0.001	0.925–0.982	0.3	91.0	90.5
	T	0.898	<0.001	0.856–0.939	538.0 ms	72.1	93.2
	T × L	0.829	<0.001	0.773–0.885	0.87 s^2^	66.4	86.5
School age	T/L	0.947	<0.001	0.898–0.996	0.3	91.6	88.9
	T	0.907	<0.001	0.849–0.966	527.8 ms	77.1	92.6
	T × L	0.859	<0.001	0.785–0.932	0.82 s^2^	71.1	88.9
	dis-D	0.686	0.004	0.577–0.794	2280.2 ms	61.4	70.4
	L	0.657	0.015	0.541–0.772	1398.8 ms	79.5	51.9
Adolescence	T/L	0.942	<0.001	0.892–0.991	0.3	82.1	95.7
	T	0.873	<0.001	0.800–0.946	466.3 ms	87.2	74.5
	T × L	0.807	<0.001	0.716–0.899	0.87 s^2^	64.1	85.1
	pro-D	0.681	0.004	0.569–0.794	773.7 ms	87.2	46.8

*p < 0.05 indicates statistical significance. AUC, area under the curve; CI, confidence interval; dis-D, the distance between the origin and the distal end of the longitudinal axis; L, longitudinal axis; ms, millisecond; POTS, postural tachycardia syndrome; pro-D, the distance between the origin and the proximal end of the longitudinal axis; s, second; T, transverse axis; T/L, the ratio of transverse axis value to longitudinal axis value; T × L, the product of transverse axis value and longitudinal axis value; VVS, vasovagal syncope*.

### Validation of Differential Diagnostic Performance Using T/L of Poincaré Plot in Validation Study

Eighty-eight children (40 boys and 48 girls; median age, 11.5 years old with a range of 6.0–16.0 years old), including 39 children with clinical diagnosis of VVS and 49 children with clinical diagnosis of POTS, were included for the validation study. The HUTT was performed for all the 39 patients with VVS, and all were positive in accordance with haemodynamic types of VVS, with a median positive reaction time after tilt of 29.0 min (range, 4.0–45.0 min). Thirty-two patients (82.1%) were categorized as the vasoinhibitory type, and 7 (17.9%) as the mixed type. The HUTT was performed in 43 of the 49 patients with POTS, among whom 13 showed a positive response of POTS pattern and the other 30 showed a negative response. However, in the standing test, the patients with a negative response during HUTT and the 6 patients who did not undergo HUTT had a positive response indicating POTS. For children with POTS, the peak heart rate was (122 ± 12) bpm and median increase of heart rate from the recumbent to orthostatic position was 43 bpm (IQR, 41 bpm −53 bpm) during the standing test or within the first 10 min of HUTT.

The T/L of the Poincaré plot, which showed the maximum AUC value in the above ROC curve analysis, was selected as the indicator for distinguishing VVS from POTS in the validation study. According to the cut-off value derived from the above analysis, with a T/L > 0.3, children were deemed to be diagnosed with VVS; otherwise, POTS was diagnosed. The accuracy, sensitivity, and specificity of T/L of the Poincaré plot in distinguishing VVS from POTS were 81.8, 87.2, and 77.6%, respectively, in the school-aged children and the adolescents; 76.5, 82.6, and 71.4%, respectively, in the school-aged children; and 89.2, 93.8, and 85.7%, respectively, in the adolescents, by comparing the T/L-based diagnosis with the clinical diagnosis (Table 5).

**Table 5:** Validation of differential diagnostic performance of T/L in Poincaré plot in validation study.

**T/L-based diagnosis** ^a^		**Clinical diagnosis**, ***n*** **(%)**	
**Age stratification**	**Groups**	**VVS**	**POTS**
School age and adolescence	VVS (*n* = 45)	34 (87.2%)	11 (22.4%)
	POTS (*n* = 43)	5 (12.8%)	38 (77.6%)
School age	VVS (*n* = 27)	19 (82.6%)	8 (28.6%)
	POTS (*n* = 24)	4 (17.4%)	20 (71.4%)
Adolescence	VVS (*n* = 18)	15 (93.8%)	3 (14.3%)
	POTS (*n* = 19)	1 (6.2%)	18 (85.7%)

a *By T/L cut-off of 0.3. POTS, postural tachycardia syndrome; T/L, the ratio of transverse axis value to longitudinal axis value; VVS, vasovagal syncope*.

## Discussion

We explored the role of the Poincaré plot derived from a 24-h Holter recording in distinguishing VVS from POTS in pediatric patients. Our study showed that the morphological properties of the Poincaré plot were significantly different between children with VVS and those with POTS. In the school-aged children, T, L, T/L, T × L, and dis-D values of patients with VVS were greater than those of patients with POTS; and in the adolescents, T, T/L, T × L, and pro-D values of patients with VVS were greater than those of patients with POTS. Among these parameters, the T/L of the Poincaré plot was found to have the most ideal discriminatory ability. Using the T/L cut-off value of 0.3 to distinguish VVS from POTS, the sensitivity and specificity were 91.0 and 90.5%, respectively, for the total participants; 91.6 and 88.9%, respectively, for the school-aged children; and 82.1 and 95.7%, respectively, for the adolescents in the derivation study. Our external validation study verified that the accuracy, sensitivity, and specificity of T/L with a cut-off value of 0.3 in distinguishing VVS from POTS were 81.8, 87.2, and 77.6%, respectively, for the total participants; 76.5, 82.6, and 71.4%, respectively, for the school-aged children; and 89.2, 93.8, and 85.7%, respectively, for the adolescents. In other words, for children who were suspected to be patients with VVS or POTS, those with the T/L of Poincaré plot > 0.3 were more likely to have VVS, and those with the T/L of Poincaré plot ≤ 0.3 were more likely to be diagnosed with POTS.

The Poincaré plot based on a 24-h Holter can reflect the distribution of the RR intervals throughout the whole day in a two-dimensional graph; therefore, it comprehensively and intuitively demonstrates the HRV, and this can indicate the tone of autonomic nervous system (21, 30). The width of the Poincaré plot graph, which was represented by T in our study, was found to be proportional to the vagal activity, while the length of the Poincaré plot graph, which was represented by L in our study, was reported to be inversely proportional to the sympathetic activity (30). Accordingly, the T/L ratio can be taken as an indicator to assess the interaction or balance between vagal and sympathetic activities (21, 30). Some studies have proposed that the imbalance between vagal and sympathetic tone was involved in the pathogenesis of VVS and POTS. Most results supported that patients with VVS had relatively high vagal activity (16, 31–33), while overactivation of sympathetic activity was one aspect of the pathogenesis of POTS (17, 34, 35). The differences in the autonomic nervous activities between VVS and POTS in children are the theoretical basis for using the Poincaré plot to distinguish them. We found that the T/L of the Poincaré plot had favorable discriminatory performance when differentiating VVS from POTS with a cut-off value of 0.3, and this was confirmed by the validation study. The superiority of T/L in its discriminatory ability may be explained by the fact that T/L can comprehensively reflect both vagal and sympathetic tone. In addition, we found that patients with VVS were younger than patients with POTS in the derivation study, which reflected the natural distribution of the age of patients during the study period. Accordingly, the height and weight of children with VVS were also lower than those of children with POTS. According to the Expert Consensus Statement of Heart Rhythm Society in 2015, the incidence of VVS begins to increase remarkably around the age of 11 and most patients with POTS present with symptoms between the ages of 15 and 25 years old (36). An investigation from Canadian adults (*n* = 443) showed that the most common age at symptom onset for VVS was 13 years old (37), and a large cross-sectional study included both children and adults (*n* = 4835) in the USA showed that the peak age at symptom onset in patients with POTS was 14 years old (38, 39). It seems that the age at onset of VVS is slightly earlier than that of POTS. However, there are few large-sample epidemiological studies comparing the age at onset of VVS and that of POTS in the same population, especially in childhood. Therefore, further studies in this regard are warranted. Finally, the positive diagnostic rate of POTS in standing test was higher than that of HUTT. One reason may be that, during the patients' hospitalization, the standing test was performed more than once (twice or three times, at most) for those who showed negative results in their first standing tests, while the tilt test was performed only once regardless of the result. Another possible reason may be that active standing and passive tilting are not equivalent maneuvers and it has been reported that active standing test was more sensitive for provoking POTS than HUTT (40, 41).

VVS and POTS are the two main entities of pediatric OI, representing acute OI and chronic OI respectively. The differential diagnosis between the two diseases is directly related to the further treatment of diseases and the evaluation of prognosis (42). Clinical history is crucial in the differential diagnosis (2). Children with VVS often experience intermittent episodes of syncope or presyncope and remain asymptomatic between the episodes; whereas children with POTS often experience chronic, non-episodic symptoms, including light-headedness, hearing changes, or fatigue (1, 7). As shown in the comparison of baseline characteristics in Table 1, children with POTS were more likely to have day-to-day symptoms as well as more frequent episodes of OI symptoms compared to those with VVS. Moreover, symptoms of VVS and POTS sometimes overlap (6). The HUTT can help in distinguishing between the two conditions (2); however, as mentioned earlier, it is associated with some flaws and risks (8–11). Therefore, it makes sense to explore other indicators to assist in differential diagnosis of VVS and POTS. The index T/L obtained from the Poincaré plot achieved good differential diagnostic performance when evaluated using clinical history and HUTT, indicating that it may be a useful index for the correct diagnosis. Compared with previous studies on differential diagnosis between VVS and POTS (13–15, 18), the graphic parameters of the Poincaré plot have obvious advantages as mentioned above. In addition, the highest sensitivity and specificity of previous indicators associated with HRV for differentiating VVS from POTS were 73.3 and 72.5%, respectively (18), both of which were lower than those of T/L in this study. These results suggest that the Poincaré plot may be superior to the frequency-domain indexes of HRV in comparing the autonomic nervous activities between children with VVS and POTS. Moreover, the autonomic functions in children gradually develop and mature with age (43). This study considered age as a factor and a stratified analysis was implemented to ensure that the results were accurate. Furthermore, the Poincaré plot can be easily obtained after a patient undergoes a 24-h Holter monitoring, and the T/L of the Poincaré plot can be intuitively seen according to the shape of the graph. That is, the ‘tennis racket' pattern tends to indicate VVS, and the ‘baseball bat' pattern tends to indicate POTS, and this can be simply observed and conveniently applied in primary hospitals. Finally, when using the Poincaré plot to distinguish VVS from POTS, pediatricians can simultaneously understand the autonomic nervous functional status of patients, which would be helpful for individualized treatment based on the pathogenesis. To the best of our knowledge, this is the first study to investigate the role of the Poincaré plot in making a differential diagnosis of OI in children. We believe that our findings indicate a new field for the application of the Poincaré plot.

Our study has several limitations, including the single-center nature and relatively small sample size. In addition, we excluded patients with VVS and POTS to obtain the indicator with the best differential diagnostic efficiency and avoid confounding results. The discriminatory performance of Poincaré plot needs to be further investigated in patients with both VVS and POTS. Finally, we did not assess other indicators related to autonomic dysfunction such as vitamin B12 levels for all the participants (44, 45). Thus, in the future, large-sample-sized and multi-center-based prospective studies are needed to further verify the value of the Poincaré plot in distinguishing VVS from POTS in children in clinical practice.

The graphic parameters of the Poincaré plot are significantly different between VVS and POTS in pediatric patients, and the T/L of the Poincaré plot has considerable differential diagnostic power. For pediatric patients with a suspicious diagnosis of VVS or POTS, those with a T/L of > 0.3 and a tennis racket-shaped Poincaré plot are more likely to be diagnosed with VVS, and pediatric patients with a T/L of ≤ 0.3 and a baseball bat-shaped Poincaré plot are more likely to be diagnosed with POTS. The T/L of the Poincaré plot may be a useful indicator to help distinguish VVS from POTS in children and adolescents.

## Data Availability Statement

The raw data supporting the conclusions of this article will be made available by the authors, without undue reservation.

## Ethics Statement

The study was approved by the Ethics Committee of Peking University First Hospital [Number of Ethics Review: (2021) Science Research No. 150] and performed according to the Declaration of Helsinki. Since it was a retrospective study and all the data, which did not include any private information of the patients, were collected from the medical records system, the requirement for informed consent was waived by the Ethics Committee of Peking University First Hospital.

## Author Contributions

PY contributed to the patient enrolment, data collecting, the statistical analysis of the study and drafting the manuscript. ZL assisted with analyzing the data and revising the manuscript. YrW, YyW, and CZ contributed to the head-up test or head-up tilt test operation. JD and YH gave important advice on the subject, designed the study, and revised the manuscript. YL supervised the design and the execution of the study, checked the data analysis, and contributed to the writing and revising of the manuscript. All authors have read and approved the final manuscript and assumed full responsibility for its contents.

## Funding

This work was supported by the Peking University Clinical Scientist Program (BMU2019LCKXJ001) and the Fundamental Research Funds for the Central Universities.

## Conflict of Interest

The authors declare that the research was conducted in the absence of any commercial or financial relationships that could be construed as a potential conflict of interest.

## Publisher's Note

All claims expressed in this article are solely those of the authors and do not necessarily represent those of their affiliated organizations, or those of the publisher, the editors and the reviewers. Any product that may be evaluated in this article, or claim that may be made by its manufacturer, is not guaranteed or endorsed by the publisher.

## Abbreviations

AUC: area under the curve
BP: blood pressure
dis-D: distance between the origin and the distal end of the longitudinal axis
HRV: heart rate variability
HUTT: head-up tilt test
L: longitudinal axis
OI: orthostatic intolerance
POTS: postural tachycardia syndrome
pro-D: distance between the origin and the proximal end of the longitudinal axis
ROC: receiver operating characteristic
T: transverse axis
VVS: vasovagal syncope.
