# Supplementary material for: Poincaré Plot Is Useful for Distinguishing Vasovagal Syncope From Postural Tachycardia Syndrome in Children
Source: Front Pediatr. 2022 Mar 10;10:758100. doi: 10.3389/fped.2022.758100 (PMC8965582; doi:10.3389/fped.2022.758100)
Supplement: Supplementary file 1 [file Data_Sheet_1.doc]

Supplementary Material

**SUPPLEMENTAL TABLE 1 Reproductivity of graphic parameters of Poincaré plot measurement between different operators**

| Subject number | T, ms | |  | L, ms | |  | T/L | |  | T×L, s^2^ | |  | pro-D, ms | |  | dis-D, ms | |
| --- | --- | --- | --- | --- | --- | --- | --- | --- | --- | --- | --- | --- | --- | --- | --- | --- | --- |
|  | Operator 1 | Operator 2 |  | Operator 1 | Operator 2 |  | Operator 1 | Operator 2 |  | Operator 1 | Operator 2 |  | Operator 1 | Operator 2 |  | Operator 1 | Operator 2 |
| Subject 1 | 358.7 | 348.4 |  | 1455.2 | 1455.2 |  | 0.2 | 0.2 |  | 0.52 | 0.51 |  | 819.9 | 819.9 |  | 2275.1 | 2275.1 |
| Subject 2 | 573.9 | 573.9 |  | 1373.2 | 1383.5 |  | 0.4 | 0.4 |  | 0.79 | 0.79 |  | 830.1 | 819.9 |  | 2203.3 | 2203.3 |
| Subject 3 | 563.6 | 553.4 |  | 1701.2 | 1690.9 |  | 0.3 | 0.3 |  | 0.96 | 0.94 |  | 850.6 | 850.6 |  | 2551.7 | 2541.5 |
| Subject 4 | 563.6 | 563.6 |  | 1475.7 | 1465.4 |  | 0.4 | 0.4 |  | 0.83 | 0.83 |  | 840.4 | 840.4 |  | 2316.1 | 2305.8 |
| Subject 5 | 819.9 | 809.6 |  | 1742.2 | 1731.9 |  | 0.5 | 0.5 |  | 1.43 | 1.40 |  | 850.6 | 850.6 |  | 2592.8 | 2582.5 |
| Subject 6 | 563.6 | 553.4 |  | 1926.6 | 1916.4 |  | 0.3 | 0.3 |  | 1.09 | 1.06 |  | 737.8 | 737.8 |  | 2664.4 | 2654.2 |
| Subject 7 | 297.2 | 297.2 |  | 1291.2 | 1291.2 |  | 0.2 | 0.2 |  | 0.38 | 0.38 |  | 830.1 | 830.1 |  | 2121.3 | 2121.3 |
| Subject 8 | 276.7 | 287.0 |  | 1127.2 | 1137.5 |  | 0.2 | 0.3 |  | 0.31 | 0.33 |  | 727.6 | 727.6 |  | 1854.9 | 1865.1 |
| Subject 9 | 297.2 | 307.5 |  | 1240.0 | 1250.2 |  | 0.2 | 0.2 |  | 0.37 | 0.38 |  | 830.1 | 830.1 |  | 2070.1 | 2080.3 |
| Subject 10 | 348.4 | 358.7 |  | 1342.5 | 1342.5 |  | 0.3 | 0.3 |  | 0.47 | 0.48 |  | 727.6 | 727.6 |  | 2070.1 | 2070.1 |
| Subject 11 | 327.9 | 327.9 |  | 1485.9 | 1496.2 |  | 0.2 | 0.2 |  | 0.49 | 0.49 |  | 881.3 | 881.3 |  | 2367.2 | 2377.5 |
| Subject 12 | 338.2 | 327.9 |  | 1475.7 | 1465.4 |  | 0.2 | 0.2 |  | 0.50 | 0.48 |  | 922.3 | 932.5 |  | 2398.0 | 2398.0 |
| Subject 13 | 266.4 | 256.2 |  | 1404.0 | 1393.7 |  | 0.2 | 0.2 |  | 0.37 | 0.36 |  | 748.1 | 748.1 |  | 2152.1 | 2141.8 |
| Subject 14 | 358.7 | 358.7 |  | 1096.5 | 1096.5 |  | 0.3 | 0.3 |  | 0.39 | 0.39 |  | 686.6 | 686.6 |  | 1783.1 | 1783.1 |
| Subject 15 | 379.2 | 368.9 |  | 1557.7 | 1557.7 |  | 0.2 | 0.2 |  | 0.59 | 0.57 |  | 922.3 | 932.5 |  | 2480.0 | 2490.2 |
| Subject 16 | 440.7 | 430.4 |  | 1516.7 | 1506.4 |  | 0.3 | 0.3 |  | 0.67 | 0.65 |  | 840.4 | 850.6 |  | 2357.0 | 2357.0 |
| Subject 17 | 430.4 | 430.4 |  | 1824.1 | 1803.6 |  | 0.2 | 0.2 |  | 0.79 | 0.78 |  | 768.6 | 778.8 |  | 2592.8 | 2582.5 |
| Subject 18 | 563.6 | 563.6 |  | 1629.4 | 1619.2 |  | 0.3 | 0.3 |  | 0.92 | 0.91 |  | 686.6 | 686.6 |  | 2316.0 | 2305.8 |
| Subject 19 | 512.4 | 522.6 |  | 1670.4 | 1660.1 |  | 0.3 | 0.3 |  | 0.86 | 0.87 |  | 789.1 | 789.1 |  | 2459.5 | 2449.2 |
| Subject 20 | 768.6 | 830.1 |  | 1567.9 | 1567.9 |  | 0.5 | 0.5 |  | 1.21 | 1.30 |  | 778.8 | 789.1 |  | 2346.7 | 2357.0 |
| Paired samples | r = 0.996 | |  | r = 1.000 | |  | r = 0.995 | |  | r = 0.996 | |  | r = 0.997 | |  | r = 0.999 | |
| Correlation | p <0.01 | |  | p <0.01 | |  | p <0.01 | |  | p <0.01 | |  | p <0.01 | |  | p <0.01 | |
| Paired *t* test | p = 0.781 | |  | p = 0.090 | |  | p = 0.514 | |  | p = 0.870 | |  | p = 0.104 | |  | p = 0.418 | |

Dis-D, the distance between the origin and the distal end of the longitudinal axis; L, longitudinal axis; ms, millisecond; pro-D, the distance between the origin and the proximal end of the longitudinal axis; s, second; T, transverse axis; T/L, the ratio of transverse axis value to longitudinal axis value; T×L, the product of transverse and longitudinal axes values.

**SUPPLEMENTAL TABLE 2 Results of measurement (two trials) of the graphic parameters of Poincaré plot in 20 subjects**

| Subject number | T, ms | |  | L, ms | |  | T/L | |  | T×L, s^2^ | |  | pro-D, ms | |  | dis-D, ms | | | |
| --- | --- | --- | --- | --- | --- | --- | --- | --- | --- | --- | --- | --- | --- | --- | --- | --- | --- | --- | --- |
|  | First time | Second time |  | First time | Second time |  | First time | Second time |  | First time | Second time |  | First time | Second time |  | | First time | Second time |  |
| Subject 1 | 358.7 | 348.4 |  | 1455.2 | 1455.2 |  | 0.2 | 0.2 |  | 0.52 | 0.51 |  | 819.9 | 819.9 |  | | 2275.1 | 2275.1 |  |
| Subject 2 | 573.9 | 584.1 |  | 1373.2 | 1383.5 |  | 0.4 | 0.4 |  | 0.79 | 0.81 |  | 830.1 | 830.1 |  | | 2203.3 | 2213.6 |  |
| Subject 3 | 563.6 | 563.6 |  | 1701.2 | 1690.9 |  | 0.3 | 0.3 |  | 0.96 | 0.95 |  | 850.6 | 850.6 |  | | 2551.7 | 2541.5 |  |
| Subject 4 | 563.6 | 553.4 |  | 1475.7 | 1465.4 |  | 0.4 | 0.4 |  | 0.83 | 0.81 |  | 840.4 | 840.4 |  | | 2316.1 | 2305.8 |  |
| Subject 5 | 819.9 | 830.1 |  | 1742.2 | 1742.2 |  | 0.5 | 0.5 |  | 1.43 | 1.45 |  | 850.6 | 850.6 |  | | 2592.8 | 2592.8 |  |
| Subject 6 | 563.6 | 563.6 |  | 1926.6 | 1916.4 |  | 0.3 | 0.3 |  | 1.09 | 1.08 |  | 737.8 | 737.8 |  | | 2664.4 | 2654.2 |  |
| Subject 7 | 297.2 | 287.0 |  | 1291.2 | 1291.2 |  | 0.2 | 0.2 |  | 0.38 | 0.37 |  | 830.1 | 830.1 |  | | 2121.3 | 2121.3 |  |
| Subject 8 | 276.7 | 276.7 |  | 1127.2 | 1137.5 |  | 0.2 | 0.2 |  | 0.31 | 0.31 |  | 727.6 | 727.6 |  | | 1854.9 | 1865.1 |  |
| Subject 9 | 307.5 | 297.2 |  | 1240.0 | 1250.2 |  | 0.2 | 0.2 |  | 0.38 | 0.37 |  | 830.1 | 830.1 |  | | 2070.1 | 2080.3 |  |
| Subject 10 | 348.4 | 348.4 |  | 1342.5 | 1352.8 |  | 0.3 | 0.3 |  | 0.47 | 0.47 |  | 727.6 | 727.6 |  | | 2070.1 | 2080.4 |  |
| Subject 11 | 327.9 | 327.9 |  | 1485.9 | 1485.9 |  | 0.2 | 0.2 |  | 0.49 | 0.49 |  | 881.3 | 881.3 |  | | 2367.2 | 2367.2 |  |
| Subject 12 | 338.2 | 338.2 |  | 1475.7 | 1475.7 |  | 0.2 | 0.2 |  | 0.50 | 0.50 |  | 922.3 | 922.3 |  | | 2398.0 | 2398.0 |  |
| Subject 13 | 266.4 | 256.2 |  | 1404.0 | 1404.0 |  | 0.2 | 0.2 |  | 0.37 | 0.36 |  | 748.1 | 740.9 |  | | 2152.1 | 2144.9 |  |
| Subject 14 | 358.7 | 358.7 |  | 1096.5 | 1106.8 |  | 0.3 | 0.3 |  | 0.39 | 0.40 |  | 686.6 | 686.6 |  | | 1783.1 | 1793.4 |  |
| Subject 15 | 379.2 | 389.4 |  | 1557.7 | 1557.7 |  | 0.2 | 0.3 |  | 0.59 | 0.61 |  | 922.3 | 922.3 |  | | 2480.0 | 2480.0 |  |
| Subject 16 | 440.7 | 440.7 |  | 1516.7 | 1527.0 |  | 0.3 | 0.3 |  | 0.67 | 0.67 |  | 840.4 | 840.4 |  | | 2357.0 | 2367.3 |  |
| Subject 17 | 430.4 | 430.4 |  | 1824.1 | 1824.1 |  | 0.2 | 0.2 |  | 0.79 | 0.79 |  | 768.6 | 768.6 |  | | 2592.8 | 2592.8 |  |
| Subject 18 | 563.6 | 563.6 |  | 1629.4 | 1629.4 |  | 0.3 | 0.3 |  | 0.92 | 0.92 |  | 686.6 | 686.6 |  | | 2316.0 | 2316.0 |  |
| Subject 19 | 522.6 | 522.6 |  | 1670.4 | 1680.7 |  | 0.3 | 0.3 |  | 0.87 | 0.88 |  | 789.1 | 789.1 |  | | 2459.5 | 2469.7 |  |
| Subject 20 | 778.8 | 778.8 |  | 1567.9 | 1578.2 |  | 0.5 | 0.5 |  | 1.22 | 1.23 |  | 778.8 | 768.6 |  | | 2346.7 | 2346.8 |  |
| Paired samples | r = 0.999 | |  | r = 1.000 | |  | r = 0.999 | |  | r = 0.999 | |  | r = 0.999 | |  | | r = 1.000 | |  |
| Correlation | p <0.01 | |  | p <0.01 | |  | p <0.01 | |  | p <0.01 | |  | p <0.01 | |  | | p <0.01 | |  |
| Paired *t* test | p = 0.491 | |  | p = 0.134 | |  | p = 0.139 | |  | p = 0.956 | |  | p = 0.169 | |  | | p = 0.319 | |  |

Dis-D, the distance between the origin and the distal end of the longitudinal axis; L, longitudinal axis; ms, millisecond; pro-D, the distance between the origin and the proximal end of the longitudinal axis; s, second; T, transverse axis; T/L, the ratio of transverse axis value to longitudinal axis value; T×L, the product of transverse and longitudinal axes values.
